# Brain and Spinal Cord Adaptations Associated With Patellofemoral Pain: A Systematic Review and Meta-Analysis

**DOI:** 10.3389/fnint.2022.791719

**Published:** 2022-02-07

**Authors:** Kai-Yu Ho, Jing Nong Liang, Savanna Budge, Austin Madriaga, Kara Meske, Derrick Nguyenton

**Affiliations:** Department of Physical Therapy, University of Nevada, Las Vegas, Las Vegas, NV, United States

**Keywords:** patellofemoral pain, spinal cord, H-reflex, brain, corticospinal excitability, neurophysiological adaptation, cortical reorganization

## Abstract

**Objective:**

To evaluate the evidence for altered cortical and spinal cord functions in individuals with patellofemoral pain (PFP).

**Methods:**

We conducted a comprehensive search of databases to appraise and analyze the studies published prior to December 10, 2021 that examined spinal reflex excitability measured using Hoffmann reflex (H-reflex) amplitudes, corticospinal excitability measured using transcranial magnetic stimulation (TMS)-elicited motor evoked potential (MEP) amplitudes, motor threshold (MT), or stimulus-response (SR) curves, cortical reorganization assessed using TMS cortical mapping or structural magnetic resonance imaging (MRI), or functional changes of the brain assessed using functional MRI (fMRI) in individuals with PFP.

**Results:**

Eight studies were eligible for analyses. While an earlier study showed that pain had no effect on the H-reflex amplitude of the quadriceps muscle, more recent evidence reported a decrease in vastus medialis (VM) H-reflex amplitude in participants with PFP. VM H-reflex amplitude was correlated with pain, chronicity, physical function, and isometric knee extensor torque production in participants with PFP. Altered corticospinal excitability was reported in participants with PFP, observed as increased MT in the VM and vastus lateralis (VL) muscles. In addition, cortical reorganization has been observed, where decreased number of cortical peaks, shifts and reduced volumes, and increased overlap of motor cortex representations for the VM, VL, and rectus femoris (RF) muscles were reported in participants with PFP.

**Conclusion:**

There is emerging evidence on altered cortical and spinal cord functions in individuals with PFP, however, solid conclusions cannot be drawn due to limited literature available. Further research is needed to better understand the adaptations of the brain and spinal cord in this population.

**Systematic Review Registration:**

https://www.crd.york.ac.uk/prospero/, identifier: CRD42020212128.

## Introduction

Patellofemoral pain (PFP) is prevalent throughout the lifespan, affecting not only the general population but also specific populations such as adolescents, highly active individuals, and the military, with an incidence rate of 9–15% (Smith et al., 2018). Furthermore, females are 2.23 times more likely to experience PFP than males, with a prevalence of 12–13% in those ages 18–35 years (Roush and Curtis Bay, 2012). One hallmark symptom of PFP is pain around or behind the patella, which is often exacerbated by loading of the patellofemoral joint in a flexed knee position (Crossley et al., 2016; Collins et al., 2018).

Individuals with PFP often exhibit difficulty performing weight-bearing tasks such as negotiating stairs, squatting, and running (Crossley et al., 2016). For example, an increase in dynamic knee valgus is a common movement deficit observed during those functional movements in this population (Powers et al., 2017; Scholtes and Salsich, 2017). This atypical pattern is the result of excessive hip adduction and internal rotation, which causes excessive loading to the lateral aspect of the patella and PFP (Powers et al., 2017; Scholtes and Salsich, 2017). As weakness of hip musculature (i.e., hip abductors and hip external rotators) is believed to attribute to excessive knee valgus during weight-bearing activities (Powers et al., 2017), addressing hip strength deficits is a commonly theorized treatment for such faulty movements (Willy et al., 2012). However, while hip strengthening programs have been shown to reduce pain and hip weakness deficits, the evidence supporting hip strengthening on improving dynamic knee valgus during functi.onal activities is limited (Willy et al., 2019; Davis et al., 2020).

In fact, as neuromuscular control is essential while performing functional movements, it has been found that a gait retraining protocol effectively corrects the frontal plane movement deficits during running in participants with PFP while a hip muscle strengthening program alone does not (Davis et al., 2020). These gait retraining protocols often incorporate motor learning principles, such as faded feedback and external-focus feedback designs (Willy et al., 2012; Davis et al., 2020). In addition, the skill of maintaining proper movements was found to be transferable to unlearned tasks, such as squatting and stair descent (Willy et al., 2012). Recent literature supports the significant role of corticomotor excitability in hip kinematics during weight-bearing activities in asymptomatic participants (Shih et al., 2021). These findings highlight the needs for examining the role of the brain and spinal cord in individuals with PFP, as the neural mechanisms underlying altered motor control in this population remain poorly understood.

Supraspinally, the motor cortex plays a critical role in motor output, and altered motor cortex structure and function underlie movement dysfunction in individuals with PFP. Motor evoked potential (MEP) amplitudes of the quadriceps muscle elicited using transcranial magnetic stimulation (TMS) revealed altered corticomotor control in participants with chronic PFP compared to asymptomatic participants (On et al., 2004). Furthermore, chronic PFP has been reported to induce reorganization of the primary motor cortex, with shifts and reduced volumes in motor representations of three quadriceps muscles, and increased overlap of the motor cortex representations, compared to asymptomatic participants (Te et al., 2017). At the level of the spinal cord, the Hoffmann reflex (H-reflex) is a commonly used electrophysiological measure to quantify the excitatory behavior of the monosynaptic Ia afferent volleys in the spinal cord circuitry. This assessment of the Ia afferent-motoneuronal pathway has been used to investigate the role and transmission of the spinal circuitry underlying motor control, and its adaptations in movement disorders, lesions or training (Schieppati, 1987; Pierrot-Deseilligny and Burke, 2012; Liang and Brown, 2015; Liang et al., 2019). Female participants with chronic PFP had significantly lower H-reflex amplitudes in the vastus medialis (VM) muscle as well as lower patellar tendon reflexes compared to asymptomatic participants. Furthermore, the altered H-reflex amplitudes were strongly associated with pain levels, where females with PFP who had larger H-reflexes amplitudes in the VM muscle had lower pain (de Oliveira Silva et al., 2016, de Oliveira Silva et al., 2017; Pazzinatto et al., 2019). Understanding the pathological mechanisms underlying PFP is thus important for future design of rehabilitation protocols targeting neural control underlying movement dysfunction in this population. Taken together, the objective of this systematic review was to evaluate the evidence for altered cortical and spinal cord functions in individuals with PFP.

## Methods

### Protocol

The protocol for conducting the review was prepared using the Preferred Reporting Items for Systematic Review and Meta-Analysis Protocols guidelines (PRISMA-P) (Shamseer et al., 2015), and has previously been peer-reviewed and published (Liang et al., 2021). Additionally, this systematic review protocol was registered with the International Prospective Register of Systematic Reviews (PROSPERO) (Registration number: CRD42020212128).

### Inclusion and Exclusion Criteria

This systematic review included relevant articles that fulfilled the following criteria: (1) original research, (2) available in full-text, (3) written in English, (4) had clear diagnostic criteria for PFP, and (5) measured at least one of the following variables in participants with PFP: (1) spinal reflex excitability measured using H-reflex amplitudes, (2) corticospinal excitability measured using TMS-elicited MEP amplitudes, motor threshold (MT), or stimulus-response (SR) curves, (3) cortical reorganization assessed using TMS cortical mapping or structural magnetic resonance imaging (MRI), and (4) functional changes of the brain using functional MRI (fMRI). Case studies, case series, and articles focused on lower extremity injuries unrelated to PFP, were excluded.

### Search Strategy

A literature search for records published prior to December 10, 2021 was conducted using the following databases: PubMed, Medline via OVID, Embase, and Web of Science. Search strings and medical subject headings (MeSH) keywords related to the themes of PFP, and non-invasive assessments of brain and spinal cord functions were used. Search strings included (“patellofemoral pain” OR patellofemoral pain syndrome” OR “anterior knee pain” OR “PFP”) AND (“reciprocal inhibition” OR “corticospinal” OR “corticospinal excitability” OR “spinal reflex” OR “spinal excitability” OR “stretch reflex” OR “Hoffmann^*^ reflex” OR “H-reflex” OR “H:M ratio” OR “primary motor cortex” OR “motor evoked potential^*^” OR “MEP^*^” OR “motor threshold” OR “cortex reorganization” OR “transcranial magnetic stimulation” OR “TMS” OR “neural excitability” OR “mapping” OR “magnetic resonance imaging” OR “functional magnetic resonance imaging”). The asterisks indicated any potential suffix for each respective term and were input into each database.

**Figure 1:**
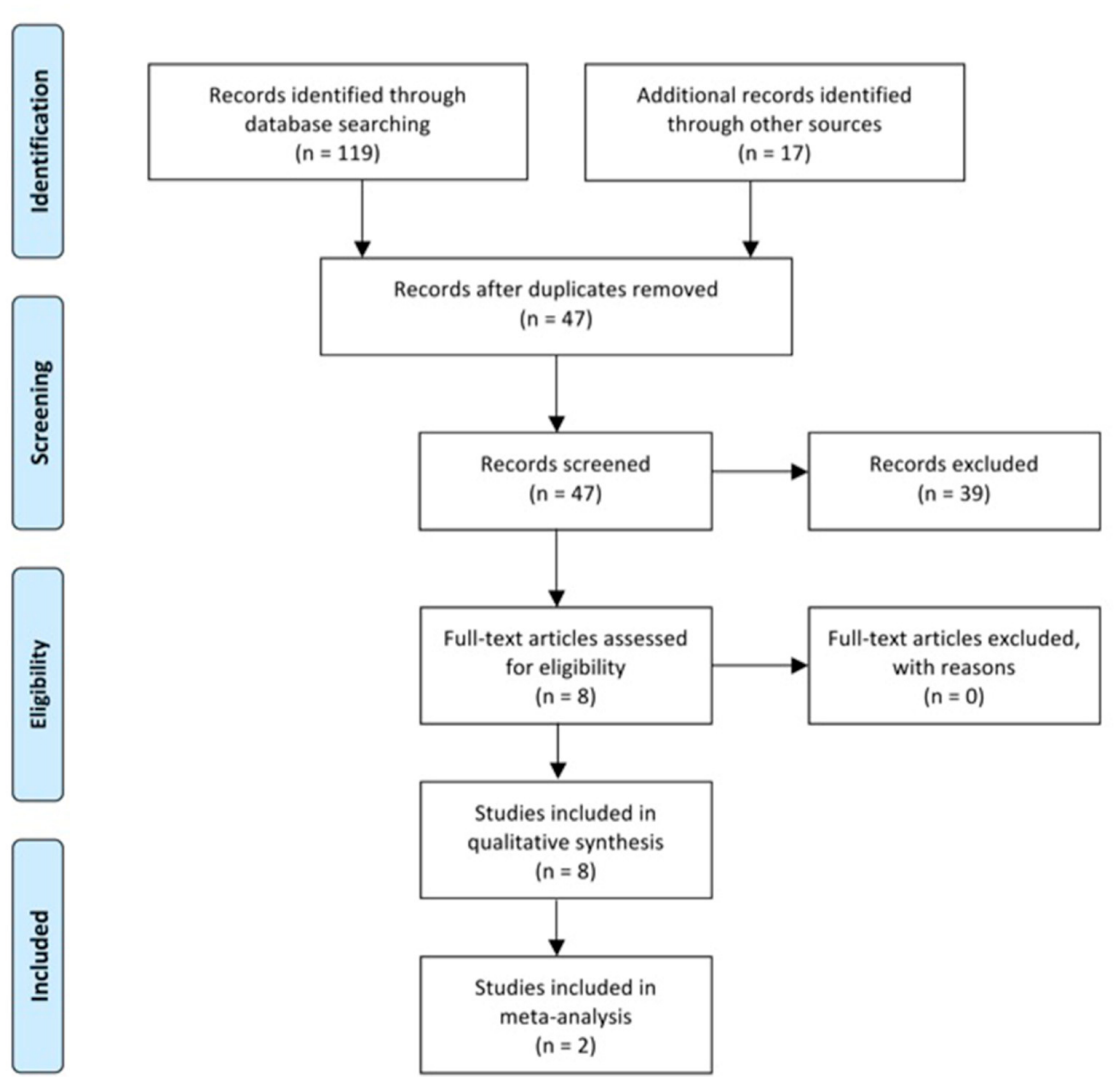
Flow diagram of PRISMA depicting each step of study selection.

Four investigators (SB, AM, KM, and DN) manually searched and inspected the literature published before December 10, 2021. The detailed flow chart for study selection is shown in Figure 1. Specifically, the titles of the articles from database searches were initially identified for relevance and reviewers manually screened the reference list of each title for additional articles. This was followed by screening of the abstracts of all titles yielded. If abstracts were not published in English, indicated the record was not an original research article, did not have full-text, or did not include H-reflex, MEP amplitudes, MT, SR curves, MRI, fMRI, cortical reorganization or mapping as a primary outcome variable, these records were removed from the study. The full-texts of records passing the abstract screening were then read in their entirety to determine their eligibility using the inclusion and exclusion criteria. Records that lacked an abstract or had an abstract with insufficient information were also read in their entirety and assessed using the inclusion and exclusion criteria. Methods for conducting this systematic review were developed using the Guidelines for Meta-Analysis and Systematic Reviews of Observational Studies (Stroup et al., 2000).

### Assessment of Methodological Quality Across Eligible Studies

Quality of eligible studies were analyzed utilizing the Quality Assessment Tool for Observational Cohort and Cross Sectional Studies from the National Institutes of Health (NIH^1^) by four investigators (SB, AM, KM, and DN). The assessment tool included 14 questions, and responses were either yes, no, not applicable (NA), or not reported (NR). A score of 0 was given for “no” and 1 for “yes.” The methodological quality for each study was then categorized subjectively by the assessors as good, fair, or poor, in reference to previous literature (Alfuraydan et al., 2020), as the NIH Quality Assessment Tool for Observational Cohort and Cross-sectional Studies does not have cut-off ranges for scoring. The investigators independently scored each study and reconvened at a later date and compared results. Any discrepancies in the quality rating between investigators were discussed and adjusted accordingly.

### Assessment of Risks of Bias Across Eligible Studies

Risks of bias were conducted using the Cochrane risk-of-bias tool (Higgins et al., 2011) by two investigators (K-YH and JL). We assessed 8 items, including (1) Random sequence generation (selection bias), (2) Allocation concealment (selection bias), (3) Blinding of participants and personnel (performance bias), (4) Blinding of outcome assessment (detection bias): self-reported outcomes, (5) Blinding of outcome assessment (detection bias): objective measures, (6) Incomplete outcome data (attrition bias), (7) Selective reporting (reporting bias), and (8) No asymptomatic control group (other bias). The response options are low risk of bias, unclear risk of bias, high risk of bias, and NA. If the item was NR it was graded as unclear risk, and if the item was NA it was answered with NA. The investigators independently scored each study and reconvened at a later date to compare results. Any discrepancies in the risk-of-bias rating between investigators were discussed and adjusted accordingly.

### Assessment of Quality of Evidence

Quality of evidence was assessed by two investigators (K-YH and JL) using the Grading of Recommendations Assessment, Development and Evaluation (GRADE) approach (Higgins and Green, 2011). We evaluated three clusters of literature, including spinal reflex excitability, corticospinal excitability, and cortical reorganization. The investigators independently scored each category and reconvened at a later date to compare results. Any discrepancies in the evaluations between investigators were discussed and adjusted accordingly. If a category contained only one study that did not allow between-study comparisons, the analysis of evidence quality was not performed (de Oliveira Silva et al., 2020).

### Meta-Analysis

Available data were synthesized via meta-analysis using Review Manager (RevMan5, The Cochrane Collaboration). As the effect sizes of the eligible studies were assumed to represent a random sample from a particular distribution of effect sizes, a random-effects model was used (Borenstein et al., 2010; Pigott and Polanin, 2019). *P* < 0.05 was considered statistically significant.

## Results

### Literature Search, Study Selection, and Study Characteristics

A total of 119 records were identified through database search. After duplicates were removed, the abstracts of 30 studies were screened to determine further evaluation. Of the 30 abstracts screened, 17 new records were identified from the reference lists and the abstracts of these records were screened with the same criteria. All of the 47 records had abstracts available for the abstract screening process. Out of the total 47 records in the abstract screen process, 39 were disqualified as the articles were not published in English (*n* = 1), were not an original research article (n=8), did not have full-text (*n* = 1), or did not use H-reflex, MEP amplitudes, MT, SR curves, MRI, fMRI, cortical reorganization or mapping as a primary outcome variable (*n* = 32). Three articles were disqualified for more than one reason. Eight studies met the eligibility criteria and of those, 5 reported H-reflex, 2 reported MEP amplitude and MT, and 1 reported cortical reorganization. None of the qualified records reported MRI or fMRI. Figure 1 shows the PRISMA flowchart for literature search and selection. The participant information for each study is summarized in Table 1.

**Table 1:** Study characteristics and participant demographics for included studies.

**References**	**Age in year; mean (standard deviation)**	**Sample size**	**Sex**	**Symptom duration in month; mean (standard deviation)**
Pazzinatto et al., 2019	Control: 22.47 (3.19) PFP: 21.83 (3.35)	60 (30 controls; 30 PFP)	Female	52.7 (56.4)
de Oliveira Silva et al., 2016	Control: 23.67 (3.75) PFP: 22.07 (3.17)	30 (15 controls; 15 PFP)	Female	66.2 (12.5)
de Oliveira Silva et al., 2017	PFP: 22.07 (3.17)	15 PFP	Female	66.2 (12.5)
Waiteman et al., 2022	PFP: 21.71 (3.30)	24 PFP	Female	50.04 (51.75)
Leroux et al., 1995	Not reported. Age range for control and PFD: 21–40	12 (6 controls; 6 PFP)	Not Reported	Not Reported
On et al., 2004	Control: 25.1 (7.4) PFP: 25 (8.1)	26 (13 controls; 13 PFP)	Female	37.9 (22.8)
Rio et al., 2016	Control: 26 [median] PFP: 26.5 [median]	18 (8 control; 10 AKP)	Both; Male >Female	90 [median]
Te et al., 2017	Control: 21 (7) PFP: 24 (6)	22 (11 controls; 11 PFP)	Both; Female > Male	31.5 (29.2)

*PFP, patellofemoral pain; PFD, patellofemoral dysfunction; AKP, anterior knee pain*.

### Methodological Quality and Risks of Bias

**Figure 2:**
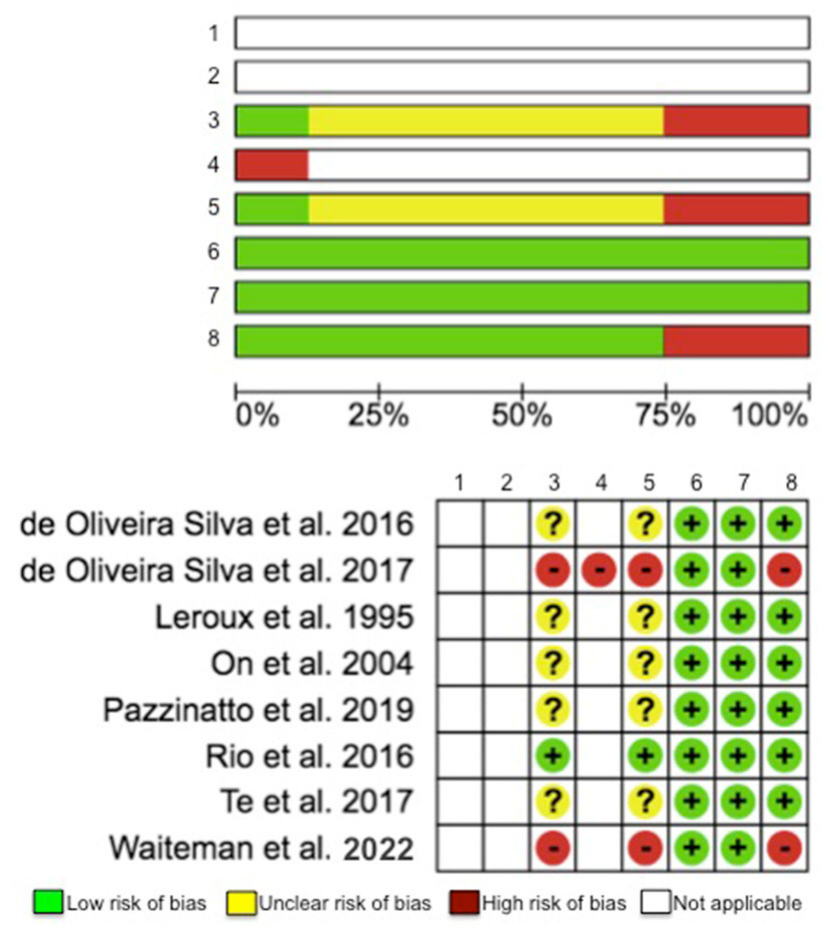
Risk-of-bias assessment for evaluating following items: (1) Random sequence generation (selection bias), (2) Allocation concealment (selection bias), (3) Blinding of participants and personnel (performance bias), (4) Blinding of outcome assessment (detection bias): self-reported outcomes, (5) Blinding of outcome assessment (detection bias): objective measures, (6) Incomplete outcome data (attrition bias), (7) Selective reporting (reporting bias), and (8) No asymptomatic control group (other bias).

The total quality assessment scores of the articles ranged from 6 to 8 (Table 2). The maximum possible quality assessment score was 14. Based on the details reported by the articles and the total scores calculated, it was determined that the study quality was similar (fair) across the studies (Alfuraydan et al., 2020). The analysis of risks of bias of the articles revealed two primary sources of bias, including blinding of participants and personnel and blinding of outcome assessment: objective measures (25% high risk and 62.5% unclear risk). Among the 8 included studies, only one study clearly stated that blinding procedure was used in their work. Given that the studies included in this review were all cross-sectional, observational studies, the first 2 items regarding participant randomization and allocation were rated as NA (Figure 2).

**Table 2:** Quality assessment of the articles using the National Institutes of Health Quality Assessment Tool for Observational Cohort and Cross Sectional Studies.

**Study**	**Q1**	**Q2**	**Q3**	**Q4**	**Q5**	**Q6**	**Q7**	**Q8**	**Q9**	**Q10**	**Q11**	**Q12**	**Q13**	**Q14**	Total score^*^	**Quality score**
Pazzinatto et al., 2019	1	1	NR	1	1	0	0	NA	1	NA	1	1	NA	1	8	Fair
de Oliveira Silva et al., 2016	1	1	NR	1	1	0	0	NA	1	NA	1	NR	NA	1	7	Fair
de Oliveira Silva et al., 2017	1	1	NR	1	1	0	0	NA	0	NA	1	NA	NA	1	6	Fair
Waiteman et al., 2022	1	1	NR	1	1	0	0	NA	0	NA	1	NA	NA	1	6	Fair
Leroux et al., 1995	1	1	NR	0	1	0	0	NA	1	1	1	NR	NA	NR	6	Fair
On et al., 2004	1	1	NR	1	0	0	0	NA	1	NA	1	NR	NA	1	6	Fair
Rio et al., 2016	1	1	NR	1	1	0	0	NA	1	NA	1	1	NA	NR	7	Fair
Te et al., 2017	1	1	NR	1	1	0	0	NA	1	NA	1	NR	NA	1	7	Fair

*NR, not reported; NA, not applicable; 1, yes; 0, no*.

* *Score out of a total possible of 14*.

### Neurophysiological Changes in PFP

#### Spinal Reflex Excitability

In total, 5 articles were analyzed with a cumulative sample size of 141 participants. The results of these articles are summarized in Table 3. Three out of the 5 studies compared participants with PFP to asymptomatic participants (Leroux et al., 1995; de Oliveira Silva et al., 2016; Pazzinatto et al., 2019), and the remaining two studies included participants with PFP only (de Oliveira Silva et al., 2017; Waiteman et al., 2022). The quality of evidence of this category was rated as low credibility (Table 4).

**Table 3:** Primary outcomes for all included studies.

**Study**	**Groups**	**Summary of data**
**Spinal reflex excitability**		
Pazzinatto et al., 2019	PFP vs. asymptomatic	• Females with PFP had lower VM H-reflex amplitude [PFP: 0.10 (0.08) %Mmax vs. asymptomatic: 0.25 (0.20) %Mmax] and patellar tendon reflex amplitude [PFP: 0.14 (0.09) %Mmax vs. asymptomatic: 0.23 (0.16) %Mmax]. • VM H-reflex and patellar tendon reflex were strongly correlated in both groups (PFP: *r* = 0.66, *p* < 0.001; asymptomatic: *r* = 0.72; *p* < 0.001).
de Oliveira Silva et al., 2016	PFP vs. asymptomatic	• Females with PFP had lower VM Hmax/Mmax ratio (14% lower) compared to asymptomatic. • VM Hmax/Mmax ratios (sensitivity = 73%, specificity = 67%) can be used to discriminate between females with and without PFP.
de Oliveira Silva et al., 2017	PFP only (no asymptomatic)	• Negative correlation between VM H-reflex amplitudes and 2 variables: worst pain in the previous month (*r* = −0.71; *p* = 0.003) and chronicity (*r* = −0.71; *p* = 0.001). • Positive correlation between H-reflex amplitudes and self-reported functional status (*r* = 0.62; *p* = 0.012).
Waiteman et al., 2022	PFP only (no asymptomatic)	• A moderate relationship between lower VM Hmax/Mmax ratio and higher variability of submaximal knee extensor torque production (*r* = −0.35, *p* = 0.05). • A moderate positive relationship between lower VM Hmax/Mmax ratio and lower maximal isometric knee extensor strength (*r* = 0.37, *p* = 0.044).
Leroux et al., 1995	PFD (before vs. after cold application)	• No difference in H-reflex amplitudes of VM, VL, and RF in participants with PFD before and after cold application for pain relief.
**Corticospinal excitability**		
On et al., 2004	PFP vs. asymptomatic	• Greater MEP amplitudes of the VMO and VL, and smaller patellar tendon reflexes in the PFP compared to asymptomatic group.
Rio et al., 2016	AKP vs. asymptomatic	• No difference in maximal MEP amplitudes [AKP: 48.73 (14.34), asymptomatic: 57.26 (18.56); *p* = 0.51], active MT [AKP:, asymptomatic: 42 (7.9); *p* = 0.06] and SR curve slopes [AKP: 6.67 (2.00), asymptomatic: 6.02 (1.54)] of the RF muscle between AKP and asymptomatic groups.
**Cortical reorganization**		
Te et al., 2017	PFP vs. asymptomatic	• Participants with PFP had: ° Reduced map volumes [VM: PFP: 4.9 (1.9) cm^2^ × mV, asymptomatic: 8.7 (3.2) cm^2^ × mV; VL: PFP: 5.3 (2.0) cm^2^ × mV, asymptomatic: 8.7 (3.4) cm^2^ × mV, RF: PFP: 7.1 (1.9) cm^2^ × mV, asymptomatic: 10.8 (4.5) cm^2^ × mV; *p* < 0.05], ° Decreased number of discrete cortical peaks [VM: PFP: 1.55 (0.69), asymptomatic: 2.27 (1.35); VL: PFP: 1.46 (0.69), asymptomatic: 2.64 (1.12); RF: PFP: 1.72 (0.79), asymptomatic: 1.73 (0.90); *p* < 0.05], ° Greater overlap of M1 representations (CoG separation) (RF andVL: PFP: 0.33 (0.17) cm, asymptomatic: 0.62 (0.26) cm; RF and VM: PFP: 0.46 (0.26) cm, asymptomatic: 0.53 (0.42) cm; VL and VM: PFP: 0.27 (0.14) cm, asymptomatic: 0.50 (0.49) cm; *p* < 0.05), ° Anterior-shifted location of M1 (CoG antero-posterior location) of the 3 muscles [VM: PFP: 4.3 (0.6) cm, asymptomatic: 3.8 (1.5) cm; VL: PFP: 4.3 (0.5) cm, asymptomatic: 3.8 (1.4) cm; RF: PFP: 4.4 (0.6) cm, asymptomatic: 3.7 (1.3) cm; *p* < 0.05].

*PFP, patellofemoral pain; PFD, patellofemoral dysfunction; AKP, anterior knee pain; H-reflex, Hoffmann reflex; Hmax, maximal Hoffmann reflex; Mmax, maximal M-wave; MEP, motor evoked potential; MT, motor threshold; SR, stimulus-response; VM, vastus medialis; VMO, vastus medialis oblique; VL, vastus lateralis; RF, rectus femoris; CoG, Center of Gravity; M1, primary motor cortex*.

**Table 4:** GRADE (Grading of Recommendations Assessment, Development and Evaluation) rating.

	**Study limitations (risk of bias)**	**Indirectness**	**Inconsistency**	**Imprecision**	**Publication bias**	**GRADE conclusion**
Spinal reflex excitability	Yes	No	Yes	Yes	No	Low credibility
Corticospinal excitability	Yes	No	Yes	Yes	No	Low credibility
Cortical reorganization	–	–	–	–	–	–

*GRADE Working Group grades of evidence: high credibility, further research is very unlikely to change our confidence in the estimate of effect; moderate credibility, further research is likely to have an important impact on our confidence in the estimate of effect and may change the estimate; low credibility, further research is very likely to have an important impact on our confidence in the estimate of effect and is likely to change the estimate; very low credibility, we are very uncertain about the estimate*.

Pazzinatto et al. (2019) reported lower VM H-reflex and patellar tendon reflex amplitudes in females with PFP compared to asymptomatic participants. The VM H-reflex and patellar tendon reflex amplitudes were strongly correlated in both the PFP (*r* = 0.66; *P* < 0.001) and the asymptomatic groups (*r* = 0.72; *P* < 0.001). Similarly, de Oliveira Silva et al. (2016) observed decreased VM H-reflex amplitudes (expressed as H_max_/M_max_ ratio) in females with PFP compared to asymptomatic participants. The VM H_max_/M_max_ ratio was observed to have high sensitivity (73%) and specificity (67%) for distinguishing between participants with PFP and asymptomatic participants (de Oliveira Silva et al., 2016). In one study that had no asymptomatic group to serve as controls, conducted by de Oliveira Silva et al. (2017), VM H-reflex amplitude was positively correlated with self-reported function, and was negatively correlated with pain and chronicity in females with PFP. In the other study without asymptomatic participants as controls, conducted by Waiteman et al. (2022), VM H-reflex amplitude negatively correlated with variability of submaximal knee extensor torque production, and positively correlated with maximal isometric knee extensor strength in female participants with PFP.

Leroux et al. (1995) examined the effects of cold application for knee pain relief on quadriceps muscle H-reflex amplitudes in participants with patellofemoral dysfunction (PFD) and asymptomatic participants. Ten minutes of ice for pain relief did not affect VM, vastus lateralis (VL), and rectus femoris (RF) H-reflex amplitudes in both groups (Leroux et al., 1995).

**Figure 3:**
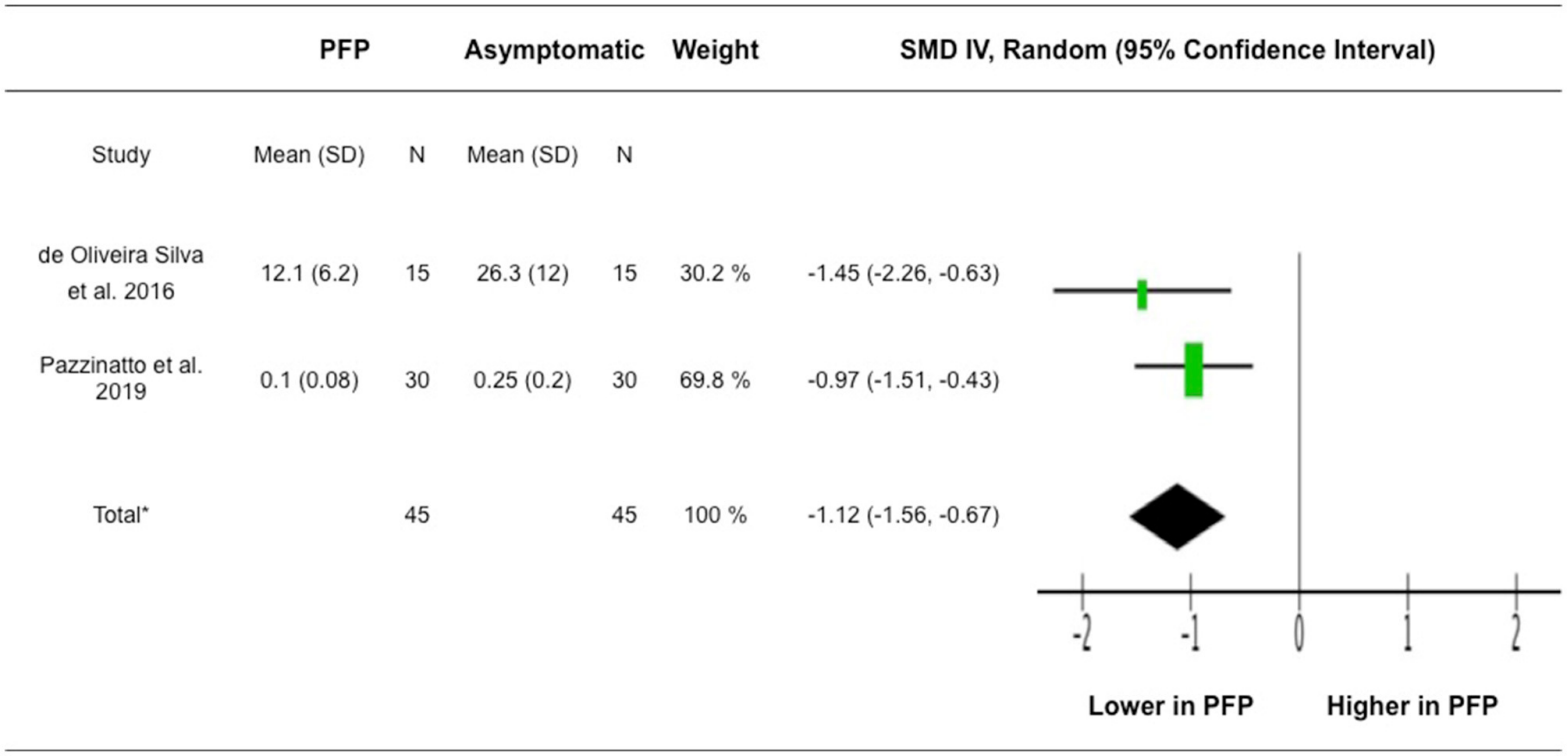
Meta-analysis for VM H-reflex amplitude. *Heterogeneity: Tau^2^ = 0.00, Chi^2^ = 0.91, df = 1 (*P* = 0.34), I^2^ = 0%. Test for overall effect: *Z* = 4.87 (*P* < 0.00001). IV, inverse variance; SMD, standardized mean difference.

Two studies that compared VM H-reflex amplitudes between participants with PFP and asymptomatic group were included for meta-analysis (de Oliveira Silva et al., 2016; Pazzinatto et al., 2019). The analysis included 90 participants. The pooled result showed significant lower VM H-reflex amplitudes associated with PFP (*z* = 4.87, 95% CI = −1.56 to −0.67, *p* < 0.001) (Figure 3).

#### Corticospinal Excitability

We included one article that examined changes in MEP amplitudes (On et al., 2004), and one article that examined active MT and SR curves, both elicited using TMS (Rio et al., 2016) in participants with PFP and anterior knee pain (AKP), respectively, compared with asymptomatic participants (Table 3). The quality of evidence of this category was considered low credibility (Table 4).

On et al. (2004) examined 26 total participants (13 females with PFP and 13 asymptomatic females). This study examined the vastus medialis oblique (VMO), VL and extensor digitorium brevis (EDB) muscles, using the amplitude of MEP as an indicator of corticospinal excitability. Greater MEP amplitudes of the VMO and VL muscles, accompanied by smaller patellar tendon responses were observed in the PFP group than in the asymptomatic group. MEP amplitude of EDB muscle did not differ between groups (On et al., 2004).

Rio et al. (2016) examined 10 participants with AKP (6 males and 4 females) and 8 asymptomatic participants (7 males and 1 female). This study examined the RF muscle and used SR curve variables (slope and maximal MEP amplitude) and active MT to represent corticospinal excitability. No differences in maximal MEP amplitudes, SR curve slopes and active MT were observed in the RF muscle between AKP and asymptomatic groups (Rio et al., 2016).

#### Cortical Reorganization

Only one study examined the changes in cortical reorganization using TMS to map the motor cortex (Te et al., 2017). Thus, the quality of evidence of this category was not assessed (Table 4). This study examined the RF, VL, and VM muscles in a total of 22 participants (11 participants with PFP and 11 asymptomatic participants). For all 3 muscles, smaller map volume, lower number of discrete map peaks, anteriorly shifted center of gravity and greater overlap of motor cortex representations of these muscles were observed in the PFP group compared to the asymptomatic group (Table 3).

## Discussion

To the best of our knowledge, this systematic review was the first conducted to identify the evidence and to provide a comprehensive review of studies examining changes in brain and spinal cord structure and neurophysiology in individuals with PFP. Overall, a solid conclusion regarding the changes in brain and spinal cord in individuals with PFP cannot be made due to limited evidence and/or conflicting evidence in the current available literature. Of the 8 eligible studies, 2 reported corticospinal excitability, 1 reported cortical reorganization and 5 reported spinal reflex excitability. Conflicting evidence was reported with respect to the changes in spinal reflex excitability in participants with PFP. Based on our search, there is no research that examines the associations between the corticospinal integrity or motor cortex representation of the gluteal muscles and PFP. While there was emerging evidence suggesting potential changes in brain function detected by fMRI (Diekfuss et al., 2020), this study was excluded as full-text was not available and the diagnostic criteria for PFP were not clear. Taken together, future studies are required to examine the changes in neurophysiological functions of the spinal cord and brain in individuals with PFP.

### Spinal Reflex Excitability

Five articles with an emphasis in neurophysiological changes at the level of the spinal cord were examined with a collective sample size of 141 participants (90 PFP and 51 asymptomatic). Studies examined the H-reflex amplitudes of the VM, VL, and RF muscles, in participants with chronic PFP. An earlier study conducted in 1995 examined knee pain in participants with PFD, and reported no change in VM, VL, and RF H-reflex amplitudes before and after cold application to the knee for pain relief, suggesting that pain did not have an effect on quadriceps H-reflex amplitudes in participants with knee pain from PFD (Leroux et al., 1995).

Our meta-analysis of the data extracted from 2 more recent studies (de Oliveira Silva et al., 2016; Pazzinatto et al., 2019) revealed reduced VM H-reflex amplitudes in participants with PFP compared to asymptomatic participants. It was suggested that reduced spinal reflex excitability may be a reason underlying increased pain and decreased function and muscle strength among participants with PFP. Specifically, a correlation was reported between the VM H-reflex amplitude and pain, chronicity, and function in participants with PFP, where participants with greater VM H-reflex amplitude have less pain, more recent symptoms and better physical function (de Oliveira Silva et al., 2017). The recent study conducted by Waiteman et al. (2022) further demonstrated that VM H-reflex amplitude is negatively associated with higher variability of submaximal torque production and positively associated with maximal isometric torque production of the quadriceps muscles in participants with PFP. Their findings highlight the relationship between altered H-reflex excitability and impaired modulation of motor unit firing rates and motor unit recruitment in participants with PFP.

In addition, VM H_max_/M_max_ ratio was found to have high sensitivity and specificity for discriminating between females with and without PFP (de Oliveira Silva et al., 2016). It was also reported that patellar tendon reflex correlated strongly with VM H-reflex in participants with and without PFP (Pazzinatto et al., 2019), suggesting that testing the patellar tendon reflex in a clinical setting may be beneficial for identifying a potential contributing factor to PFP (Pazzinatto et al., 2019).

### Brain Structure and Neurophysiology

Three articles with an emphasis in brain neurophysiological and structural changes were examined with a collective sample size of 66 participants (24 participants with PFP, 10 with AKP, 32 asymptomatic; On et al., 2004; Rio et al., 2016; Te et al., 2017).

#### Corticospinal Excitability

Two studies examined alterations in corticospinal excitability associated with PFP and AKP, however the two studies employed different methodology and analysis, each presenting different variables to represent corticospinal excitability. One study reported greater MEP amplitudes of VM and VL muscles in individuals with PFP elicited using TMS compared to controls (On et al., 2004). This increase in MEP suggests that there may be alterations in corticospinal excitability in this population. The other more recent study examined the RF muscle and used SR curve (slope and maximal MEP amplitude) and active MT to represent corticospinal excitability. No differences in maximal MEP amplitudes, SR curve slopes and active MT were observed in the RF muscle between AKP and asymptomatic participants (Rio et al., 2016). As there were only two studies used to examine the effects of PFP on corticospinal excitability using TMS (On et al., 2004; Rio et al., 2016), each examining different variables to represent corticospinal excitability, additional high-quality experimental studies are needed to provide stronger and more conclusive evidence to support adaptations of corticospinal tract in chronic PFP.

#### Cortical Reorganization

One study examined cortical reorganization in participants with chronic PFP, via TMS delivered to the primary motor cortex contralateral to the affected side (Te et al., 2017). Findings included smaller map volumes, a more anteriorly located center of gravity, and a lower number of cortical peaks for the VM, VL, and RF muscles, as well as greater overlap in motor cortex representations between quadriceps muscle pairs in participants with PFP compared to asymptomatic participants (Te et al., 2017).

Decreased map volumes in the quadriceps muscles were suggested to be associated with reduced neural projections from the brain to the quadriceps muscle. The authors explained that altered map volumes could also be a result of lower cortical excitability, however conflicting findings from other studies did not support this plausible explanation (On et al., 2004; Rio et al., 2016). Specifically, On et al. (2004) found that corticomotor excitability increased in the VM and VL as a result of decreased map volumes in participants with PFP, while Rio et al. (2016) suggested no changes in excitability of the RF in participants with AKP as compared to asymptomatic participants. Further research examining the relationship between MEP amplitude and decreased map volumes is required to better delineate the extent of spinal excitability changes and cortical changes in individuals with PFP (Te et al., 2017).

The increased number of high cortical peaks has been linked to the potential for complex, synergistic movements (Te et al., 2017). In addition, low cortical peaks have been hypothesized to relate to lower excitability of the corticospinal tract. This suggests that individuals with PFP may not adapt to various environments and tasks as easily as asymptomatic individuals, which may provide an explanation as to why individuals with PFP experience difficulties in performing certain functional tasks, such as stairs negotiation, single leg hops, and jumps (Te et al., 2017).

Te et al. (2017) suggested that the anterior shift of the centers of gravity of the VM, VL, and RF muscles reflected the presence of remodeling and plasticity in the primary motor cortex. Although this study did not specifically examine the changes in motor control or function, similar findings in other populations led them to suggest that the mechanism of the anterior shift is related to synaptic changes to the neurons that give input to pyramidal cells. Specifically, Shanahan et al. (2015) have found an anterior shift of motor representations of the VM, VL, and RF muscles in participants with osteoarthritis. Such associations between the shifts of the quadriceps muscle representations and poorer quadriceps performance may explain the movement disorders in participants with PFP (Te et al., 2017). As this was the only article found that examined the relationship between cortical reorganization and PFP, more research is needed to confirm and generalize results.

### Quality and Risk-of-Bias Assessment Implications

The quality assessment tool and risk-of-bias assessment tool used were beneficial for gaining an overall sense of consistency, or lack thereof, of the studies combined. The quality for the studies evaluated was considered fair, with scores between 6 and 8. The risk-of-bias analysis also revealed the key bias of the current studies in this area, such as blinding of participants and personnel, blinding of outcome measures, and no asymptomatic control group. These assessment tools can be used for future research to qualitatively address the potential risks of selection bias, detection bias, or confounding variables. For example, to optimize the study quality and reduce bias, future studies can improve by specifying inclusion and exclusion criteria for all participants, conducting a sample size analysis, implementing a blinding procedure, using asymptomatic control groups, and stating clear diagnostic criteria of PFP.

### Implications for Clinical Practices and Research

A recent systemic review highlighted the importance of patient education in the management of PFP, including understanding the anatomy of the joint, physiology of pain, activity modification, and quadriceps muscle strengthening (de Oliveira Silva et al., 2020). Our systemic review findings regarding central nervous system changes in participants with PFP highlight the need for including neurophysiological dysfunctions of the quadriceps muscles in patient education of this cohort. Furthermore, a potential intervention is to alleviate central inhibition of the affected muscles via transcranial direct current stimulation (tDCS), which is a non-invasive brain stimulation that modulates cortical excitability (Lefaucheur et al., 2017). It has been shown that a 4-week (12-session) tDCS protocol focusing on corticomotor excitation of the knee extensor musculature improved quadriceps muscle strength and symptoms in participants with PFP (Rodrigues et al., 2020).

In addition, Glaviano et al. (2019) have theorized that central inhibition of gluteal musculature is a possible mechanism of hip muscle dysfunction and faulty movement patterns in individuals with PFP, which may lead to prolonged dysfunction and pain. However, this theory has not yet been tested in individuals with PFP. Overall, most available literature examined the spinal level and cortical level separately, thus evidence for contributions and/or adaptations from spinal or descending supraspinal sources remain limited. As the existing studies examined the spinal and cortical changes utilized a cross-sectional study design, it remains unclear whether such changes are the risk factors of PFP or the consequents of chronic pain (Pazzinatto et al., 2017). Taken together, immediate future efforts should examine cortical and spinal levels concurrently to elucidate the relative contributions of supraspinal vs. spinal influences contributing to altered knee and hip muscle function. Ideally, a larger scale, longitudinal study that tracks the central nervous system adaptations in young adults until the appearance of PFP may facilitate the understanding the underlying neurophysiological mechanism of PFP.

## Conclusion

This systematic review provides the current evidence for neurophysiological brain and spinal cord changes in participants with PFP and has identified the need for further research on the corticospinal excitability and spinal reflex excitability in this population. Although there is conflicting literature surrounding the impact of PFP on spinal reflex excitability, more recent evidence supports the idea that spinal reflex excitability is decreased in individuals with PFP. Evidence of changes in brain function is inconclusive because of the differences in outcomes studied and limited literature available, but the current evidence supports that PFP may have an influence on the corticospinal tract and cortical reorganization. The need for more research on the cortical and spinal changes associated with PFP is evident to better understand viable treatments for individuals with this pathology from a neurophysiological perspective in addition to the biomechanical models currently in use.

## Data Availability Statement

The original contributions presented in the study are included in the article/supplementary material, further inquiries can be directed to the corresponding author/s.

## Author Contributions

K-YH and JL conceived and designed the study. K-YH, JL, SB, AM, KM, and DN developed the search strategies, conducted analysis of the study, and were involved in writing and editing of the manuscript. All authors approved the final manuscript.

## Funding

The work was supported by Student Opportunity Research Grant, Department of Physical Therapy, University of Nevada, Las Vegas. The publication fees for this article were supported in part by the University of Nevada, Las Vegas, MSI Graduate Student Open Access Fund.

## Conflict of Interest

The authors declare that the research was conducted in the absence of any commercial or financial relationships that could be construed as a potential conflict of interest.

## Publisher's Note

All claims expressed in this article are solely those of the authors and do not necessarily represent those of their affiliated organizations, or those of the publisher, the editors and the reviewers. Any product that may be evaluated in this article, or claim that may be made by its manufacturer, is not guaranteed or endorsed by the publisher.

## Footnotes

^1^NIH Study Quality Assessment Tools [Online]. Available online at: https://www.nhlbi.nih.gov/health-topics/study-quality-assessment-tools (accessed August 23, 2021).
